# Museum exhibition-space psychology of sequential organization and cinematic spatial cues: an exploratory mixed-model association analysis

**DOI:** 10.3389/fpsyg.2026.1939380

**Published:** 2026-10-23

**Authors:** Ziqin Yuan, Chao Zhou

**Affiliations:** 1 School of Media and Arts, Wuxi University, Wuxi, China; 2 School of Media and Design, Nantong Institute of Technology, Nantong, China

**Keywords:** cinematic spatial cues, emotional engagement, environmental psychology, museum exhibitions, narrative transportation, perceived route clarity, sequential organization, visit intention

## Abstract

This exploratory study examines whether perceived sequential organization and perceived cinematic spatial cues in ordered museum-exhibition images are statistically associated with perceived route clarity, narrative transportation, emotional engagement, and visit intention. An image-based evaluation dataset included 397 returned responses and 314 valid respondents, yielding 918 valid participant-material observations after screening. Each respondent evaluated one high-, one medium-, and one low-level stimulus set, creating the repeated participant-material structure analyzed below. Participant-random-intercept linear mixed models were used for repeated participant-material evaluations; control-adjusted, stimulus-variance-component, and participant-clustered analyses were retained as sensitivity checks. Perceived sequential organization was associated with perceived route clarity (beta = 0.358, 95% CI [0.296, 0.421]), perceived cinematic spatial cues were associated with narrative transportation (beta = 0.320, 95% CI [0.257, 0.384]), and narrative transportation was associated with emotional engagement (beta = 0.374, 95% CI [0.311, 0.436]). Perceived route clarity (beta = 0.248, 95% CI [0.184, 0.312]) and emotional engagement (beta = 0.234, 95% CI [0.170, 0.298]) were both associated with visit intention. Participant-clustered bootstrap estimates further supported the statistical indirect association from cinematic spatial cues through narrative transportation to emotional engagement (indirect effect = 0.109, 95% CI [0.079, 0.144]). The findings provide preliminary evidence of statistical associations among perceived exhibition-space cues, subjective route clarity, transportation, emotional engagement, and visit intention in image-based evaluations, without implying actual gallery movement or causal mediation.

## 1 Introduction

The functional identity of museum exhibition space is undergoing a profound shift—from “displaying objects” toward “organizing visitor experience.” Where traditional exhibition practice centered on the preservation and presentation of collections, contemporary museums increasingly emphasize how space itself can organize, guide, and shape the act of visiting (Derda, 2024). Space, on this view, is no longer a neutral vessel holding artifacts, but a medium that actively engages perception, cognition, and emotion. Recent work reinforces the point: museums are not only sites of knowledge transmission but also settings where visitors find emotional fulfillment, construct meaning, and even experience psychological wellbeing (Šveb Dragija and Jelinčić, 2022). Spatial sequence, sightline organization, rhythmic variation, concealment and revelation, the placement of key exhibits, and the overall emotional atmosphere together form the material and psychological ground on which visitor experience unfolds.

This shift resonates closely with a central concern of environmental psychology—the reciprocal relationship between people and the built environment, and particularly how built settings influence perception, cognition, emotion, and behavioral intention. The Stimulus–Organism–Response (S–O–R) framework offers a classic foundation here:environmental cues (S) are related to internal psychological states (O), which are associated with approach or avoidance responses (R) (Mehrabian and Russell, 1974). In the museum context, empirical studies have shown that architectural elements, lighting, sound, materials, and spatial organization can systematically shape visitors’ emotional experience (Zhang et al., 2024). Research on emotional responses to architectural imagery similarly indicates that people form stable emotional judgments from visualized spatial materials (Zhang et al., 2024), while work on smart art museums points to the role of environmental interaction design in regulating visitor emotion (Zhang et al., 2024). Taken together, this evidence supports a basic premise: the spatial features of a museum can be studied as measurable environmental-psychological variables.

Running parallel to the space–emotion pathway is a research tradition concerned with museum narrative and emotional connection. Studies of digital storytelling suggest that museum narratives can strengthen the emotional connection, historical empathy, and personal relevance that visitors feel toward objects, history, and cultural themes (Economou et al., 2025). Work on museum digitalization further argues that narrative is not mere information transfer, but a means—through character, situation, theme, and emotional cues—of fostering a deeper dialogue between visitors and cultural heritage (Wang and Meng, 2024). Research on exhibition narrative at a national museum likewise shows that narrative structure and modes of exhibition interaction markedly affect visitors’ learning experience and engagement (Economou et al., 2025). Narrative, in short, is widely regarded as an important driver of emotional engagement in museums.

At the level of integration, however, a clear gap remains. Space, narrative, emotion, and movement have often been examined in separate strands: space-syntax and simulation studies address how layout, path, and accessibility shape visitor movement (Wang et al., 2025), whereas online-review and machine-learning studies infer spatial preferences from indirect data (Jiang et al., 2025). Few studies have operationalized visitors’ perceptions of sequential organization and cinematic spatial cues in exhibition imagery as measurable variables and examined, within an exploratory association framework, their statistical associations with perceived route clarity, narrative transportation, emotional engagement, and visit intention. In particular, cinematic spatial cues have remained largely within architectural and design discourse, with limited effort to test them as a questionnaire-based psychological construct.

Against this background, the present study analyses a questionnaire dataset based on existing museum exhibition and gallery images. It examines how perceived sequential organization and perceived cinematic spatial cues in these images are statistically associated with perceived route clarity, narrative transportation, emotional engagement, and visit intention within an exploratory association framework. Here, perceived sequential organization refers to the perceived beginning-to-closure structure of the image sequence, while perceived cinematic spatial cues refer to framing, concealment-revelation, visual focus, spatial rhythm, and scene progression.

Because the design is image-based, the study measures perceived route clarity rather than actual movement behavior in galleries. It treats perceived sequential organization, perceived cinematic spatial cues, perceived route clarity, narrative transportation, emotional engagement, and visit intention as self-reported perceptual, experiential, and intention variables associated with visitors’ evaluations of exhibition imagery. The high-, medium-, and low-level material groups were formed through a combined theory-driven coding procedure; therefore, the design does not establish fully independent manipulations of narrative and cinematic cues. In this article, perceived route clarity refers to the inferred clarity of route direction, starting-point recognition, and spatial focus; it is a perception derived from the image sequence rather than observed movement.

Accordingly, the study asks two linked questions: (1) whether perceived sequential organization is associated with perceived route clarity; and (2) whether perceived cinematic spatial cues are associated with narrative transportation, emotional engagement, and visit intention through the specified statistical paths. These questions motivate the exploratory association analyses described in the next section.

Figure 1 summarizes the exploratory statistical-association map. The figure presents theory-informed associations among image-based perceptual appraisals, experiential responses, and visit intention; the questionnaire measures are not a direct test of the classical S–O–R model. The arrows indicate exploratory statistical associations based on image-based self-reports. H4 denotes the exploratory indirect association rather than an experimentally established causal mediation effect.

**Figure 1:**
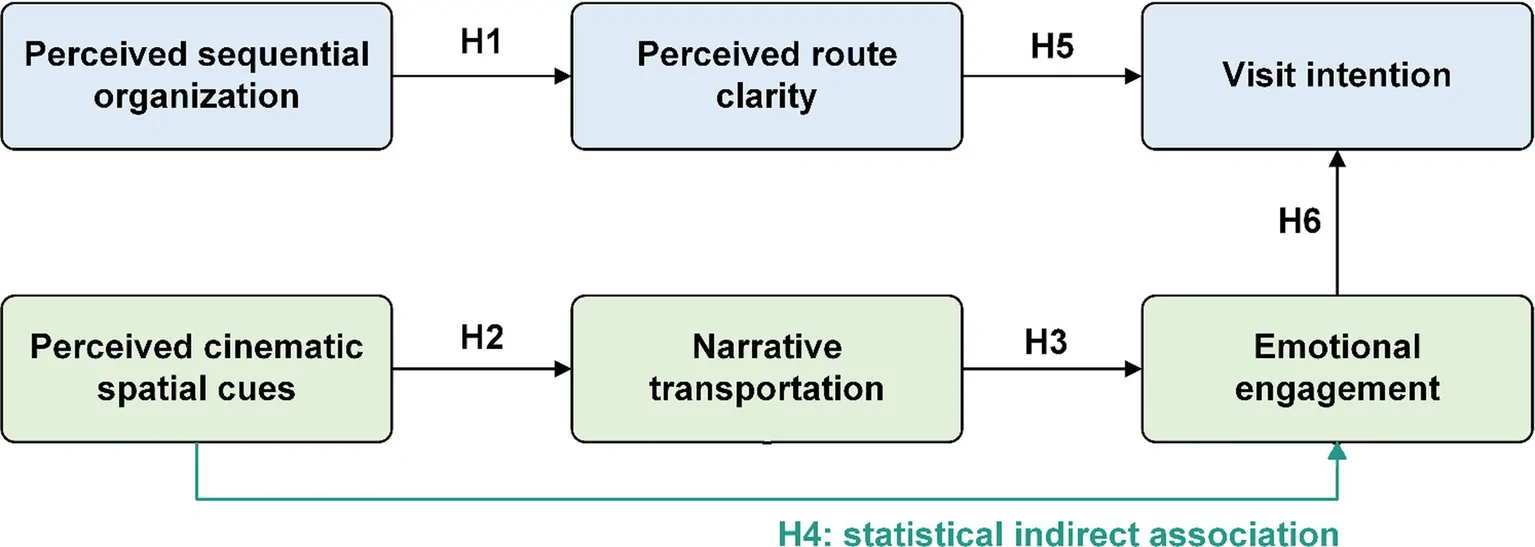
Theory-informed exploratory association map for image-based exhibition-space evaluation. The model is estimated from image-based self-reports; arrows indicate exploratory statistical associations, and H4 denotes a statistical indirect association rather than causal mediation.

## 2 Theoretical background and exploratory association expectations

### 2.1 Museum exhibition space and environmental psychology

Environmental psychology offers a systematic framework for understanding how museum exhibition space relates to visitor experience. Within this framework, the Stimulus–Organism–Response (S–O–R) model describes how environmental stimuli (S) are related to internal psychological states (O), which in turn are associated with approach or avoidance responses (R) (Mehrabian and Russell, 1974). In a museum, layout, lighting, materials, sound, and visual organization constitute potential environmental cues. Applying this framework to virtual museum experiences, prior research has linked perceived exhibition-space quality with behavioral intention through psychological variables such as emotion and cognition (Meng and Dolah, 2025). This perspective supports examining exhibition-space features as perceived environmental cues and visitor responses as psychological variables, while keeping the direction and causal status of the relationships open to empirical testing. In the present study, the ordered image sets constitute the stimulus context, whereas questionnaire scores represent post-viewing perceptual appraisals, experiential responses, and visit intention. Because objective environmental stimuli and pleasure-arousal-dominance dimensions were not measured, the study draws on S–O–R reasoning only as a general perspective and does not test the classical S–O–R model.

A substantial body of work has confirmed that museum spatial atmosphere can systematically mobilize visitors’ emotional experience. A study of the Jewish Museum Berlin found that architectural elements, lighting, sound, materials, and spatial organization are not passive backdrops but actively organize, arouse, and regulate emotional responses (Zhang et al., 2024). Experiments using editable 360-degree VR panoramas went further, showing that a single spatial parameter such as ceiling height can be enough to elicit measurable emotional differences (Zhang et al., 2023). Research on the multimodal sensory perception of museum environments indicates that visual and haptic channels jointly participate in visitors’ construction of exhibition atmosphere (Kartal and Kartal, 2026), while studies linking emotion to neural responses to architecture corroborate, at the physiological level, the stable connection between spatial features and emotional experience (Zhang et al., 2023). Together, these findings support a basic premise: visitors can form stable, measurable psychological responses from the visual features of exhibition space.

As extended reality (XR) has been adopted in the cultural-heritage field, a growing body of work has examined how emotionally oriented environmental design relates to visitor engagement. Reviews suggest that emotional design is becoming a key strategy for connecting spatial features with visitor experience (Lin et al., 2025). Much of this work, however, relies on immersive technologies or on-site deployment and therefore provides less evidence about how visitors evaluate spatial features from static imagery alone. This distinction motivates the present study’s image-based focus on perceptual evaluations of exhibition-space cues.

### 2.2 Perceived sequential organization and perceived route clarity

How museum space organizes visitor movement has long been a central question in space-syntax research. Space-syntax theory holds that the topological structure of a built space itself embodies a “spatial logic” that largely governs how people move, pause, and encounter one another within it (Hillier and Hanson, 1984). Building on this, wayfinding research points out that the visibility, accessibility, and sequentiality of a spatial configuration are decisive for whether visitors can orient themselves: clear sightlines, intuitive circulation, and hierarchical spatial transitions together form a spatial language that visitors can “read” (Peponis et al., 1990). Empirical work in open-plan exhibition settings has likewise confirmed that layout features systematically shape visitors’ spatial behavior (Peponis et al., 2004), and recent reconsiderations of the relationship between space syntax and design reaffirm the foundational role of spatial structure in organizing exploratory paths (Peponis, 2024).

In recent years, quantitative research on museum layout, path configuration, and accessibility has deepened. Studies using visibility-graph analysis and intelligent-agent simulation show that corridors and paths play a key role in organizing movement through exhibition space, which indicates that “movement” or a “sense of path” is a reasonable and measurable spatial variable (Wang et al., 2025). Analyses of the relationship between layout and visitor paths in modern museums similarly show that spatial-layout types—such as sequential versus choice spaces—directly affect how circulation forms (Medaković et al., 2024), and space-syntax examinations of natural-science museums underscore the close link between spatial permeability and wayfinding (Yue et al., 2025).

Most of this work, however, centers on actual movement in real space and pays less attention to the perceived route clarity that visitors may infer from exhibition imagery. When a sequence of exhibition images presents a clear beginning, development, focal point, and closure—that is, when it conveys a strong perceived sequential organization—viewers may form subjective judgments about the direction of the visit, the clarity of the route, and the location of key areas. Thus, a stronger perceived spatial-narrative structure is expected to be associated with greater perceived route clarity. The present study measures this perceptual judgment rather than actual visitor movement in a gallery (Wang et al., 2025). Accordingly:

*H1*: Perceived sequential organization is positively associated with perceived route clarity.

### 2.3 Cinematic spatial cues and narrative transportation

In this study, “perceived cinematic spatial cues” denotes a perceptual construct informed by work at the intersection of architecture and film. In that literature, space is often understood as a sequence experienced “frame by frame”: concealment and revelation, sightline framing, visual focus, and spatial rhythm can direct attention and create a sense of scene and image (Kafili and Brennan, 2025). These ideas provide a conceptual basis for examining whether viewers perceive cinematic qualities in exhibition-space imagery. The construct is therefore used here as a questionnaire-based psychological perception, rather than as a claim that the images reproduce film techniques or independently manipulate a distinct environmental feature. It comprises concealment–revelation, visual focus, spatial rhythm, and a sense of scene progression.

Narrative-transportation theory provides a theoretical basis for examining how perceived cinematic spatial cues may relate to visitors’ psychological experience. The theory defines transportation as an integrated state of attention, imagery, and affect—the experience of being immersed in a narrative world and temporarily leaving reality behind (Green and Brock, 2000). The transportation-imagery model further emphasizes the role of vivid mental imagery in transportation and its links with mental simulation, attitudes, and beliefs (Green and Brock, 2002). A meta-analysis across contexts likewise shows that transportation is systematically related to antecedent narrative features and to experiential and behavioral outcomes (Van Laer et al., 2014). In the present study, the “narrative” does not refer to a verbal plot or to physically entering a narrative world. It refers to the perceived progression inferred from an ordered image set, including contextual framing, transition, concealment and revelation, visual focus, spatial rhythm, and closure. These features may help viewers infer continuity and scene progression, sustain attention, evoke mental imagery, and support affective involvement, making a narrative-transportation-related association plausible. Narrative transportation is treated as distinct from immersion, presence, visual absorption, spatial interest, and emotional engagement, although these experiences may be related. In this image-based study, transportation refers to attention, mental imagery, and affective involvement inferred from an ordered image sequence.

Evidence from immersive and digital-museum research provides contextual or indirect support for the psychological plausibility of this account, but it does not directly test the present static-image pathway. Research on digital storytelling in virtual-reality museums has linked multidimensional narrative transportation—including immersion, emotional arousal, and mental imagery—with visitor–object experience (Chen et al., 2026). Studies of digital storytelling in VR exhibitions have also reported associations between narrative organization and visitors’ sense of presence and immersion (Chang and Suh, 2025). Comparisons of immersive realities (VR, VR360, and MR) report differentiated patterns of presence, engagement, and motivation across media (Ariya et al., 2025), while experiments on immersive media, emotion, and memory indicate that immersion is related to emotional experience and memory performance (Bujić et al., 2023). In addition, work treating interactive storytelling as an exhibit in its own right highlights the immersive potential of narrative within museum space (Plakhotnyuk, 2026). These findings are relevant as contextual evidence because they indicate that narrative organization, imagery, and affective involvement may matter in museum-related experience. However, they do not show that static images reproduce stereoscopic depth, embodied viewpoint, sensorimotor interaction, spatial presence, or active exploration. In the present study, these findings therefore serve as boundary evidence rather than direct validation of the association between perceived cinematic spatial cues and narrative transportation. Accordingly:

*H2*: Perceived cinematic spatial cues are positively associated with narrative transportation.

### 2.4 Narrative transportation, emotional engagement, and visit intention

Narrative transportation has also been linked to emotional engagement. In the present study, emotional engagement is treated as a multidimensional self-reported response involving emotional connection, historical empathy, personal relevance, interest, being moved, and emotional resonance (Economou et al., 2025). Studies of museum digital storytelling report that narrative and immersive experience are associated with emotional connection and empathy (Chen et al., 2026). Within transportation theory, focused attention, mental imagery, and situational involvement are proposed as psychological conditions under which emotional responses may emerge (Van Laer et al., 2014). From an embodied-cognition perspective, perception, movement, and spatial experience likewise provide contexts in which exhibition-related emotion can be formed (Long et al., 2024). Accordingly:

*H3*: Narrative transportation is positively associated with emotional engagement.

Beyond this direct link, transportation may statistically account for part of the association between cinematic spatial cues and emotional engagement. Because the dataset is cross-sectional and same-source, this hypothesis is framed as an indirect-association pattern rather than as evidence of a causal mechanism. This logic is consistent with the narrative features-transportation-experiential outcomes chain identified in the transportation-imagery model and its meta-analysis (Van Laer et al., 2014; Chen et al., 2026). Accordingly:

*H4*: Narrative transportation statistically accounts for part of the association between perceived cinematic spatial cues and emotional engagement.

For the step from experience to behavioral intention, prior research has reported links between digital museum experience and on-site visit intention through psychological variables such as cultural identity and perceived value (Deng et al., 2023). Research on virtual-museum experience quality has similarly identified associations with offline visit intention (Meng and Dolah, 2025). Studies of art-craft and cultural-heritage museums have reported relationships between visitors’ emotions, emotional engagement, satisfaction, and subsequent behavioral intentions (Pioletti et al., 2025), while research on cultural-heritage tourists has linked emotional experience with behavioral intention through variables such as place attachment and satisfaction (Li et al., 2025). Work grounded in cognition–emotion–behavior theory also highlights the statistical relevance of emotion-related variables to recommendation intention in online museums (Li et al., 2025). Related mediation analyses in museum tourism (Chen, 2024) and studies of immersive exhibition experience (Popoli and Derda, 2021) further suggest that experiential and emotional responses may be connected with visit or recommendation intention. Perceived route clarity may likewise be associated with willingness to continue a visit or attend in person: when visitors perceive the direction as clear and the route as well defined, a stronger intention to visit may be reported.

Perceived route clarity and emotional engagement may each show a distinct statistical association with visit intention, while also being correlated with one another. The present study therefore enters both variables into a single visit-intention model and estimates their associations with visit intention under mutual adjustment, rather than treating either path as an isolated causal effect. Accordingly:

*H5*: Perceived route clarity is positively associated with visit intention.

*H6*: Emotional engagement is positively associated with visit intention.

## 3 Materials and methods

### 3.1 Respondents and data structure

This was a formal online questionnaire study conducted in Chinese. Participants were recruited in mainland China through open online recruitment, primarily by forwarding a Wenjuanxing questionnaire link through the researchers’ social-network channels. The study used convenience sampling with voluntary participation. Eligibility required participants to be at least 18 years old, voluntarily consent to participate, be able to read the Chinese questionnaire, and view all museum-exhibition images in the questionnaire. No art or design background or specific museum-visiting experience was required. Data were collected from March 5 to June 5, 2026, and no monetary compensation was provided. This manuscript reports the formal questionnaire study and the theory-based stimulus-coding procedure used to organize the image materials.

The dataset contains 397 questionnaire records, corresponding to 1,191 raw participant-material observations because each respondent evaluated three stimulus groups. After questionnaire-level screening, 314 valid respondents were retained (Table 1), corresponding to 942 expected participant-material observations. After material-level attention and completeness screening, 918 valid material-level observations remained. The primary mixed models used 830–852 complete scale-score observations depending on the variables in each model, whereas the control-adjusted sensitivity models used 812–832 observations; the clustered bootstrap indirect-association models used 789–818 complete scale-score observations, and 743 rows were fully complete across all focal item-level measurement variables.

**Table 1:** Respondent characteristics and data-quality screening (*N* = 314).

Variable	Category	*n*	%
Gender	Male	141	44.9
	Female	169	53.8
	Other/prefer not to say	4	1.3
Age group	18–25	141	44.9
	26–35	105	33.4
	36–45	35	11.1
	46–55	25	8.0
	56 and above	8	2.5
Education	High school or below	19	6.1
	Junior college	46	14.6
	Bachelor’s	155	49.4
	Master’s	80	25.5
	Doctoral or above	14	4.5
Museum visit frequency	Almost never	57	18.2
	1–2 times/year	144	45.9
	3–5 times/year	82	26.1
	6 or more times/year	31	9.9
Art/design background	No	207	65.9
	Yes	107	34.1
Prior online museum use	No	118	37.6
	Yes	196	62.4

Before data collection, the study protocol specified the inclusion and exclusion criteria. Inclusion required age ≥ 18, voluntary participation, Chinese-reading ability, and complete viewing of all stimulus materials. Exclusion criteria were markedly short completion time, failed attention checks, repeated selection of the same response option (straightlining), incomplete viewing, severe missingness in key variables, and an overall response pattern judged to be random or of very low quality (random_low_quality). The last category involved 19 participants. Of 397 returned questionnaire records, 314 were retained as valid respondents (79.1%). The 83 excluded records comprised speeding (*n* = 21), random_low_quality (*n* = 19), attention-check failure (*n* = 18), straightlining (*n* = 14), incomplete viewing (*n* = 7), and excessive missingness (*n* = 4). The 314 valid respondents generated 942 expected participant-material observations; 918 remained after material-level screening.

### 3.2 Stimulus materials

The stimulus materials comprised eight candidate museum exhibition and gallery image sets selected from official online exhibition pages, virtual-tour screenshots, and open-licensed image sources. The candidate pool contained two high-sequential sets (H01 Louvre; H02 Vatican Museums), three medium-sequential sets (M03 Rijksmuseum; M01 Scottish National Gallery; M02 National Museum of China), two low-sequential/open-space sets (L02 National Gallery of Ireland; L01 The Metropolitan Museum of Art (Temple of Dendur)), and one object-heavy backup set (B01 American Museum of Natural History Northwest Coast Hall). Seven sets were retained for the formal questionnaire. The backup set was excluded because its emphasis on object display was less compatible with the required exhibition-space sequence, route continuity, and narrative progression.

Two authors, Ziqin Yuan and Chao Zhou, both of whom had training in art and design, independently coded all eight candidate stimulus sets. The coding used six prespecified theory-based indicators: clarity of starting point, sequential continuity, concealment-revelation, visual focus, spatial rhythm, and sense of closure. Each indicator was scored from 0 to 2, producing total coding scores from 0 to 12. Preliminary grouping was defined as low (0–4), medium (5–8), and high (9–12). During independent coding, each coder worked without viewing the other coder’s scores. For discrepant ratings, the coders reviewed the relevant image sequence and coding definitions item by item, discussed the discrepancy, and established a consensus coding result; if a discrepancy remained, Chao Zhou made the final adjudication under the coding rules. No formal inter-rater reliability coefficient, such as weighted Cohen’s kappa or an intraclass correlation coefficient, was calculated. The coding was used for theory-informed stimulus screening rather than as a primary outcome measure, so the final material classification is interpreted together with the participant-level manipulation checks. The procedure was not intended to claim that the image sets were equivalent in museum fame, topic, visual style, or photographic quality; these material differences are treated as limitations.

The final coding scores were as follows, in the indicator order listed above: H01 Louvre, 2/2/2/2/2/1, total 11 (high); H02 Vatican Museums, 2/2/2/2/1/1, total 10 (high); M03 Rijksmuseum, 1/2/1/2/1/1, total 8 (medium); M01 Scottish National Gallery, 1/1/1/1/1/2, total 7 (medium); M02 National Museum of China, 1/1/1/1/1/1, total 6 (medium); L02 National Gallery of Ireland, 1/1/0/1/0/1, total 4 (low); and L01 The Metropolitan Museum of Art (Temple of Dendur), 1/0/0/1/0/1, total 3 (low). The backup set B01 was excluded because it emphasized object display and had weaker spatial sequence, route continuity, and narrative progression than required for the present stimulus-screening purpose.

For article presentation, Figure 2 uses non-repeating illustrative examples to clarify the spatial-cue categories, and Figure 3 summarizes the stimulus-screening logic. Source and license metadata for the illustrative photographic panels are reported in Supplementary Table S1.

**Figure 2:**
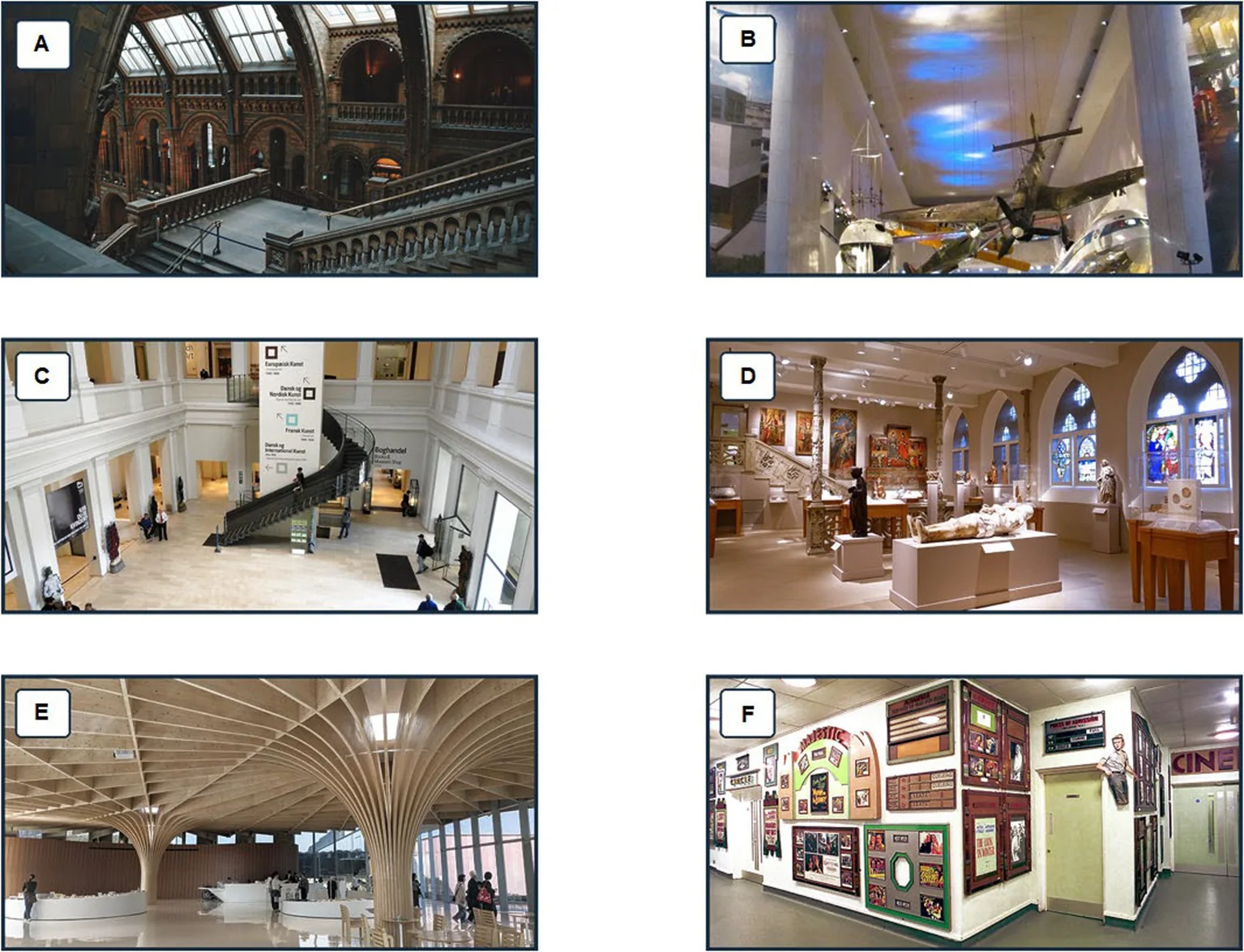
Illustrative museum exhibition-space examples showing the kinds of spatial cues discussed in the article. Photographic source and license metadata are provided in Supplementary Table S1.

**Figure 3:**
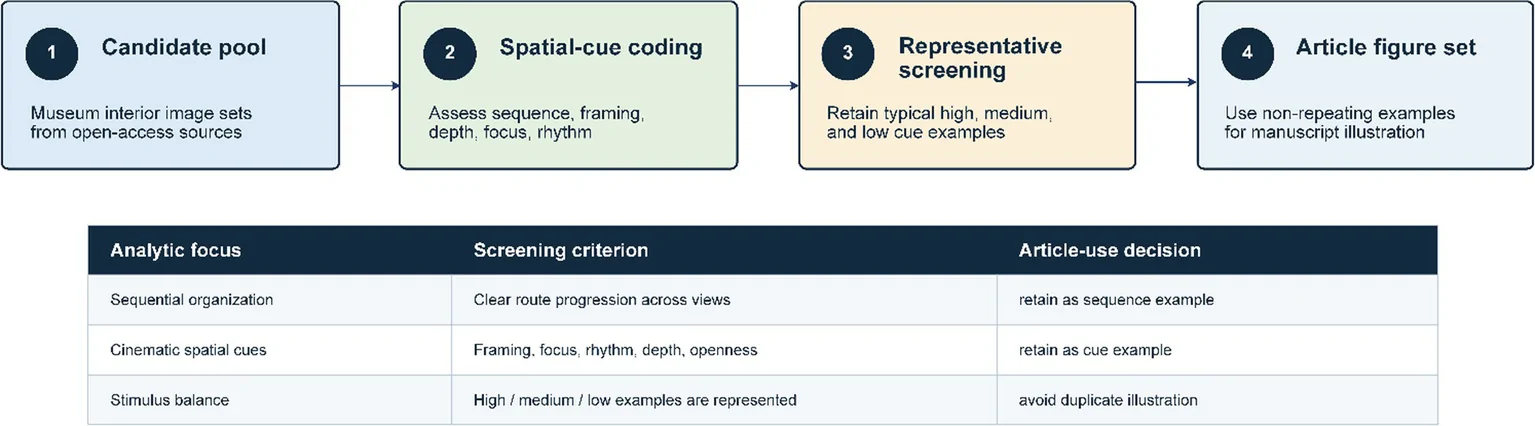
Stimulus-screening workflow used to separate spatial-cue relevance from manuscript illustration needs.

Because the actual questionnaire stimulus materials include museum website images, virtual-tour screenshots, or open-licensed images, full-resolution stimuli are not redistributed publicly owing to copyright constraints. In the paper and in data sharing, only stimulus IDs, source URLs, license and author information, metadata, coding criteria, and anonymized questionnaire data are provided. The photographic examples shown in Figure 2 are separate copyright-safe illustrative examples selected for publication display.

### 3.3 Questionnaire measures

All focal constructs were measured using items presented after each six-image stimulus sequence. Each stimulus block contained 26 evaluation items: perceived sequential organization (PSN; 4 items), perceived cinematic spatial cues (CSC; 4 items), perceived route clarity (PMG item block; 4 items), arrative transportation (NT; 4 items; adapted from Green and Brock, 2000; Appel et al., 2015), emotional engagement (EE; 4 items), visit intention (VI; 3 items), and three sensitivity covariates—topic interest, visual attractiveness, and familiarity—with one item each. All items used a 7-point Likert-type response scale ranging from 1 = strongly disagree to 7 = strongly agree. Construct scores were calculated as the arithmetic mean of the corresponding items. Higher scores indicated stronger perceived organization, spatial cues, route clarity, narrative transportation, emotional engagement, or visit intention, as applicable.

The measures were designed for the evaluation of ordered museum-exhibition images rather than for the assessment of physical movement in an actual gallery. Accordingly, perceived route clarity refers to respondents’ inferred judgments about the starting point, directional continuity, and spatial focus represented in the image sequence; it does not measure observed walking routes, dwell time, or on-site circulation. Visit intention refers to stated willingness to visit, revisit, recommend, or learn more about the represented exhibition or museum. The construct-to-item mapping and measurement sources are reported in Supplementary Table S9.

Control variables comprised interest in the exhibition topic (topic interest), the visual attractiveness of the material (visual attractiveness), and familiarity with the museum or exhibition (familiarity). Topic interest, visual attractiveness, and familiarity were treated as post-viewing sensitivity covariates rather than primary controls because they may partly reflect respondents’ immediate reactions to the same stimulus materials; including them in the primary models could therefore over-adjust the focal spatial-cue associations. Their effects are reported separately in the control-adjusted sensitivity models (Supplementary Table S2).

### 3.4 Questionnaire procedure

In the questionnaire procedure, each respondent evaluated three stimulus sets: one high-sequential, one medium-sequential, and one low-sequential set. Within each level, the available sets were H01/H02 for the high level, M01/M02/M03 for the medium level, and L01/L02 for the low level. These options generated 12 possible high-medium-low combinations, all of which were observed among the 314 respondents retained at the questionnaire level; their participant-level frequencies are reported in Supplementary Table S10. Each respondent therefore received one material from each level. The three level blocks were presented in randomized order. Each selected set contained six images displayed in the fixed sequence predefined for that set. The evaluation items and attention-check items were presented in fixed order; item randomization was not used. The complete set of realized combinations and their frequencies is reported in Supplementary Table S10. Respondent-level and material-level records were retained accordingly. The dataset included low-quality cases, mild item-level missingness, incomplete viewing records, and response-style heterogeneity, which were addressed through screening and sensitivity analysis. Before each image set, respondents were instructed to view the six images in order and to imagine moving through the space while doing so. This instruction operationalized image-guided imagined progression; it did not measure physical movement, observed routes, or dwell time in an actual exhibition space.

### 3.5 Analysis workflow

The analysis proceeded as follows. First, sample characteristics and data-quality exclusions were summarized. A *post hoc* sensitivity-oriented power interpretation followed Cohen’s effect-size guidance (Cohen, 1992). Second, participant-level manipulation checks compared the high, medium, and low material levels for PSN and CSC using participant-random-intercept mixed models because respondents evaluated repeated material sets. These checks evaluated whether participants perceived the intended graded distinction; because the material grouping was based on a combined theory-driven coding procedure, the results were not interpreted as evidence that the narrative and cinematic cues had been independently manipulated. Third, reliability and item-level measurement diagnostics were computed; the diagnostics included raw alpha, standardized alpha, PCA-based loading ranges, HTMT-style discrimination checks, and a Harman single-factor common-method diagnostic (Fornell and Larcker, 1981; Hu and Bentler, 1999; Marsh et al., 2004). Fourth, the primary hypothesis tests used participant-random-intercept linear mixed models to account for repeated participant-material observations (Baayen et al., 2008; Barr et al., 2013; Bates et al., 2015; Brauer and Curtin, 2018; Matuschek et al., 2017). Fifth, control-adjusted, stimulus-variance-component, and participant-clustered models were used as sensitivity analyses. Statistical indirect associations were estimated using participant-clustered percentile bootstrap resampling with 5,000 resamples (Preacher and Hayes, 2004; Preacher and Hayes, 2008; Hayes, 2009). Analyses were conducted in Python 3.13 using pandas, scipy, statsmodels 0.14.5, and matplotlib; compact computed sensitivity, diagnostic, sample-screening, stimulus-coding, questionnaire-item, assignment, and manipulation-check outputs are reported in Supplementary Tables S2–S11.

The repeated participant-material structure is important because each respondent evaluated multiple stimulus sets, so observations should not be treated as fully independent. The primary models included participant random intercepts. Stimulus variance components were not folded into the primary fixed-effect table because the seven-stimulus design makes stimulus-level variance sensitive to between-stimulus material differences; those estimates are instead summarized as a sensitivity diagnostic in Supplementary Table S3.

For visit intention, perceived route clarity and emotional engagement were entered into a single primary model so that each path was estimated while adjusting for the other focal predictor. Topic interest, visual attractiveness, and familiarity were treated as sensitivity-analysis variables because they may themselves be affected by stimulus viewing. Therefore, control-adjusted coefficients are reported separately rather than being mixed into the primary hypothesis table.

## 4 Results

### 4.1 Sample characteristics and manipulation check

The dataset contained 397 questionnaire records and 1,191 raw participant-material observations. After participant-level screening, 314 valid respondents remained (79.1% of returned records). The 83 excluded records comprised speeding (*n* = 21), random_low_quality (*n* = 19), attention-check failure (*n* = 18), straightlining (*n* = 14), incomplete viewing (*n* = 7), and excessive missingness (*n* = 4); the stage-by-stage sample flow is summarized in Supplementary Tables S4, S5. These respondents generated 942 expected participant-material observations; 24 material-level observations were removed because of material-level attention or completeness screening, leaving 918 valid participant-material observations. The primary mixed models used 830–852 complete scale-score observations, the control-adjusted sensitivity models used 812–832 observations, the clustered bootstrap indirect-association models used 789–818 observations, and 743 all-item-complete rows were available for same-source measurement and common-method diagnostics.

The participant-level manipulation check showed the expected graded pattern, with mean scores increasing from low to medium to high materials (Table 2). For perceived sequential organization, mean scores were 3.87 (SD = 0.82; *n* = 297), 4.82 (SD = 0.82; *n* = 291), and 5.62 (SD = 0.77; *n* = 299) for the low, medium, and high groups, respectively; the participant-random-intercept mixed model yielded Wald chi-square(2) = 833.23, *p* < 0.001, participant ICC = 0.163. For perceived cinematic spatial cues, the corresponding means were 4.01 (SD = 0.81; n = 299), 4.98 (SD = 0.83; *n* = 296), and 5.75 (SD = 0.77; *n* = 290); the mixed model yielded Wald chi-square(2) = 801.07, *p* < 0.001, participant ICC = 0.132. Thus, participants reported progressively higher levels of both perceptions across the low, medium, and high material groups. Because the levels were formed through a combined theory-driven material-coding procedure, these checks support perceived differentiation among the material groups but do not establish that narrative and cinematic cues were independently manipulated.

**Table 2:** Manipulation-check results across material levels.

Variable	Low *n*; *M* (SD)	Medium *n*; *M* (SD)	High *n*; *M* (SD)	LMM Wald chi-square (df = 2)	*p*	Participant ICC
Perceived sequential organization	*n* = 297; 3.87 (0.82)	*n* = 291; 4.82 (0.82)	*n* = 299; 5.62 (0.77)	833.23	<0.001	0.163
Perceived cinematic spatial cues	*n* = 299; 4.01 (0.81)	*n* = 296; 4.98 (0.83)	*n* = 290; 5.75 (0.77)	801.07	<0.001	0.132

Valid Ns vary slightly across variables because scale scores were computed only when all items of the corresponding scale were complete.

### 4.2 Reliability, validity, and correlations

Table 3 summarizes scale reliability and item-level measurement diagnostics; raw Cronbach alpha values ranged from 0.709 to 0.801 across the six scales. A Harman single-factor diagnostic was conducted using the item-level dataset. The first unrotated factor explained 23.35% of the total variance, below the commonly used 50% rule of thumb. This result indicates that a single dominant common factor was not apparent in the dataset, although Harman’s test is a low-sensitivity descriptive diagnostic and common method bias remains a design limitation because all focal variables were collected through the same questionnaire. Item-level HTMT-style ratios ranged from 0.155 to 0.649 (*N* = 743), below the commonly used 0.85 threshold (Supplementary Table S6), supporting discriminant separation among the focal constructs as a descriptive diagnostic. Although perceived sequential organization and perceived cinematic spatial cues were the most strongly correlated pair (HTMT = 0.649), this value remained below conservative discrimination thresholds, supporting their empirical separability in this dataset. Nevertheless, because these two spatial-cue dimensions are conceptually adjacent, future work should test competing measurement models with larger and more balanced stimulus pools.

**Table 3:** Reliability and measurement-model diagnostics.

Scale	*N*	Items	raw_alpha	std_alpha	first_eigen	mean_item_r	min_loading	max_loading	loading_range
perceived_sequential_organization	887	4	0.796	0.797	2.486	0.495	0.753	0.819	0.067
perceived_cinematic_spatial_cues	885	4	0.801	0.801	2.509	0.502	0.742	0.839	0.097
perceived_route_clarity	883	4	0.711	0.711	2.147	0.381	0.662	0.772	0.110
narrative_transportation	875	4	0.745	0.745	2.274	0.422	0.680	0.811	0.131
emotional_engagement	878	4	0.718	0.720	2.177	0.391	0.678	0.784	0.106
visit_intention	902	3	0.709	0.709	1.896	0.448	0.770	0.823	0.052

Descriptive statistics and correlations are reported in Table 4 for the valid dataset. Construct means ranged from 4.31 to 4.91 on the 7-point scale, with standard deviations from 0.92 to 1.08. The correlations were positive and ranged from *r* = 0.093 to *r* = 0.508. Because observations were nested within respondents, the correlation matrix is interpreted descriptively; participant-level and model-based analyses were used for inferential claims.

**Table 4:** Descriptive statistics and correlations in the valid dataset.

Variable	*M*	SD	1	2	3	4	5	6
1 Perceived sequential organization	4.77	1.08	0.796					
2 Perceived cinematic spatial cues	4.91	1.07	0.508	0.801				
3 Perceived route clarity	4.49	0.94	0.358	0.274	0.711			
4 Narrative transportation	4.49	0.98	0.275	0.320	0.164	0.745		
5 Emotional engagement	4.31	0.92	0.147	0.167	0.093	0.374	0.718	
6 Visit intention	4.32	1.02	0.160	0.167	0.272	0.230	0.253	0.709

Values below the diagonal are Pearson correlations computed from valid participant-material rows. Diagonal values are raw Cronbach alpha coefficients rather than Fornell–Larcker square roots of AVE; inferential interpretation should rely on the mixed models and clustered sensitivity analyses.

### 4.3 Exploratory association and indirect-association analyses

Table 5 reports the standardized main-path estimates from the primary participant-random-intercept mixed models. Control-adjusted, stimulus-variance-component, and participant-clustered sensitivity outputs are reported in Supplementary Table S3 rather than combined with the primary fixed-effect table.

**Table 5:** Linear mixed-model results (standardized fixed effects).

Hypothesis	Outcome	Predictor	beta	SE	*z*	*p*	CI low	CI high	*N* rows	Marginal *R*^2^	Conditional *R*^2^	Converged
H1	perceived_route_clarity	perceived_sequential_organization	0.358	0.0319	11.244	<0.001	0.296	0.421	852	0.128	0.155	True
H2	narrative_transportation	perceived_cinematic_spatial_cues	0.320	0.0325	9.846	<0.001	0.257	0.384	843	0.103	0.118	True
H3	emotional_engagement	narrative_transportation	0.374	0.0320	11.685	<0.001	0.311	0.436	840	0.140	0.163	True
H5/H6	visit_intention	perceived_route_clarity	0.248	0.0326	7.615	<0.001	0.184	0.312	830	0.127	0.148	True
H5/H6	visit_intention	emotional_engagement	0.234	0.0326	7.187	<0.001	0.170	0.298	830	0.127	0.148	True

H5 and H6 were estimated jointly within a single visit-intention model. The primary models include participant random intercepts and do not include post-viewing control variables. Standard errors are reported to four decimals to reduce rounding discrepancies among beta, z, and confidence intervals. Across primary models, participant variance ranged from 0.015 to 0.027, residual variance ranged from 0.837 to 0.883, total ICC values ranged from 0.016 to 0.031, marginal *R*^2^ values ranged from 0.103 to 0.140, and conditional *R*^2^ values ranged from 0.118 to 0.163 (Supplementary Table S7). Control-adjusted, stimulus-variance-component, and participant-clustered sensitivity estimates are reported in Supplementary Table S3.

The primary mixed models supported all five main statistical paths. Perceived sequential organization was positively associated with perceived route clarity, beta = 0.358, SE = 0.0319, 95% CI [0.296, 0.421], *N* = 852, marginal *R*^2^ = 0.128, conditional *R*^2^ = 0.155, supporting H1. Perceived cinematic spatial cues were positively associated with narrative transportation, beta = 0.320, SE = 0.0325, 95% CI [0.257, 0.384], *N* = 843, marginal *R*^2^ = 0.103, conditional *R*^2^ = 0.118, supporting H2. Narrative transportation was positively associated with emotional engagement, beta = 0.374, SE = 0.0320, 95% CI [0.311, 0.436], *N* = 840, marginal *R*^2^ = 0.140, conditional *R*^2^ = 0.163, supporting H3. In the joint visit-intention model, perceived route clarity, beta = 0.248, SE = 0.0326, 95% CI [0.184, 0.312], and emotional engagement, beta = 0.234, SE = 0.0326, 95% CI [0.170, 0.298], were both positively associated with visit intention, supporting H5 and H6.

Sensitivity checks showed the same directional conclusions in control-adjusted LMMs, stimulus-variance-component LMMs, and participant-clustered regressions. Because the stimulus-variance-component specification shifts much of the variance into stimulus-level differences, those estimates are treated as diagnostic rather than as the primary fixed-effect table.

As exploratory analyses, two additional statistical indirect associations were positive: perceived sequential organization → perceived route clarity → visit intention (indirect effect = 0.086, 95% CI [0.059, 0.116]), and narrative transportation → emotional engagement → visit intention (indirect effect = 0.073, 95% CI [0.042, 0.106]). These estimates were not treated as additional confirmatory hypothesis tests.

Taken together, the primary mixed models supported H1-H3, H5, and H6. The clustered bootstrap analysis was consistent with H4 as a statistical indirect-association hypothesis: the indirect association from perceived cinematic spatial cues through narrative transportation to emotional engagement was 0.109, 95% CI [0.079, 0.144]. Figure 4 reports the primary participant-random-intercept LMM fixed effects, while Table 6 reports the clustered bootstrap indirect associations.

**Figure 4:**
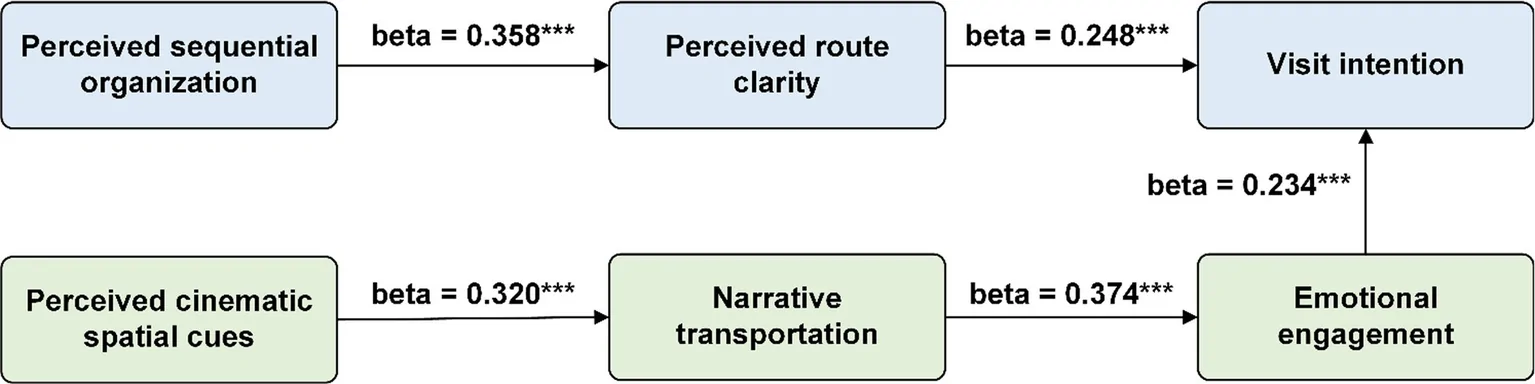
Summary of statistical associations with standardized coefficients from the primary participant-random-intercept LMMs. Arrows represent statistical paths rather than experimentally established causal effects; indirect associations are reported separately in Table 6.

**Table 6:** Participant-clustered bootstrap indirect associations (5,000 resamples).

*x*	Mediator	*y*	a_path	b_path	c_total	c_prime	indirect_effect	boot_ CI_low	boot_CI_high	N_ rows	N_participants
perceived_ cinematic_ spatial_cues	narrative_ transportation	emotional_ engagement	0.325	0.337	0.170	0.061	0.109	0.079	0.144	789	314
perceived_ sequential_ organization	perceived_ route_ clarity	visit_ intention	0.353	0.245	0.161	0.075	0.086	0.059	0.116	818	313
narrative_ transportation	emotional_ engagement	visit_ intention	0.359	0.202	0.213	0.141	0.073	0.042	0.106	808	312

Indirect effects were estimated with 5,000 participant-clustered bootstrap resamples. The b-paths are partial coefficients estimated with the independent variable included in the model, and therefore differ from the corresponding bivariate fixed effects in Table 5. For example, the emotional-engagement b-path on visit intention (0.202) is smaller than the corresponding Table 5 fixed effect (0.234) because narrative transportation is held constant in the statistical indirect-association model, so the two tables are consistent rather than conflicting.

## 5 Discussion

### 5.1 Theoretical positioning

The first theoretical contribution concerns the conceptual vocabulary for image-based exhibition-space perception. Existing environmental-psychology and museum-layout accounts have primarily emphasized configuration, visibility, accessibility, path organization, and spatial structure (Wang et al., 2025; Hillier and Hanson, 1984; Peponis et al., 1990; Peponis et al., 2004; Peponis, 2024; Medaković et al., 2024; Yue et al., 2025). The present study adds a perceptual vocabulary—framing, concealment–revelation, visual focus, spatial rhythm, and scene progression—for describing how an ordered set of exhibition images may be experienced as organized and affectively engaging. This contribution is preliminary: perceived cinematic spatial cues are a questionnaire-based perceptual construct derived from architecture–film theory, not an independently manipulated or fully validated environmental feature. Its value lies in connecting architecture–film concepts with environmental-psychological accounts at the level of perceived spatial organization (Kafili and Brennan, 2025).

The second contribution clarifies the boundary between image-based perceptual evaluation and classical environmental-psychology models. The ordered museum/gallery images constitute the stimulus context, while the questionnaire measures respondents’ perceptual appraisals, experiential responses, and visit intention. The study therefore does not directly test the classical S–O–R model, because objective environmental stimuli and the pleasure-arousal-dominance dimensions were not independently measured. This distinction prevents post-viewing questionnaire scores from being treated as if they were the environmental stimuli themselves. The cross-sectional, same-source design is consequently interpreted as an exploratory association design rather than as evidence of a temporal or causal S–O–R process.

The third contribution concerns the proposed interpretive ordering of spatial perception and narrative transportation. The results are consistent with a sequence in which viewers first infer continuity, focus, and progression from an ordered image set and may then report narrative-like involvement involving attention, mental imagery, and affect. This provides a theoretical rationale for examining the association between perceived cinematic spatial cues and narrative transportation, and for treating transportation as an experiential state potentially related to emotional engagement (Green and Brock, 2000; Green and Brock, 2002; Van Laer et al., 2014; Chen et al., 2026). The ordering is theoretical rather than empirically demonstrated: because all focal variables were collected in the same cross-sectional questionnaire, the present estimates do not establish temporal precedence or causal mediation.

Finally, the study defines a form of museum narrative that differs from conventional narrative accounts centered on curatorial text, exhibit labels, interactive storytelling, or visitor accounts. Here, narrative refers to perceived progression across an ordered set of exhibition images—contextual framing, transition, concealment and revelation, visual focus, spatial rhythm, and closure. This image-derived narrative is therefore a claim about visual representation and psychological appraisal, not about a complete narrative world or embodied gallery experience. Static images do not provide stereoscopic depth, embodied viewpoint, sensorimotor interaction, spatial presence, active exploration, or actual circulation. The theoretical contribution is consequently bounded: the study offers a preliminary account of how exhibition space may be visually organized and perceived, while claims about physical navigation remain questions for on-site, behavioral, or interactive research (Chang and Suh, 2025; Ariya et al., 2025; Bujić et al., 2023).

### 5.2 Interpretation of statistical associations

This study examined perceived sequential organization and perceived cinematic spatial cues as environmental-psychological variables and their statistical associations with perceived route clarity, narrative transportation, emotional engagement, and visit intention. Because participants evaluated exhibition imagery rather than galleries *in situ*, the findings concern image-based perceptions and intended responses; they do not establish actual movement behavior, fully independent cue manipulations, or causal mechanisms in physical galleries.

First, the H1 estimate, beta = 0.358, indicates a positive statistical association between perceived sequential organization and perceived route clarity, without implying a claim about actual walking behavior. This pattern is compatible with the possibility that clearly staged entrance, transition, focus, and closure cues help viewers form subjective judgments about route clarity when evaluating exhibition imagery.

Second, the cinematic-cue pathway was consistent with the proposed statistical sequence: perceived cinematic spatial cues were positively associated with narrative transportation, beta = 0.320, and the clustered bootstrap analysis yielded an indirect association from cinematic cues through transportation to emotional engagement of 0.109, 95% CI [0.079, 0.144]. This pattern is compatible with a transportation-related account of emotional engagement, but it does not show that cinematic cues independently caused transportation or that transportation causally produced engagement.

Third, the downstream paths formed a coherent statistical model. Narrative transportation was positively associated with emotional engagement, beta = 0.374; perceived route clarity and emotional engagement were each positively associated with visit intention in the joint model, beta = 0.248 and beta = 0.234, respectively. These estimates distinguish the observed statistical paths and joint associations from evidence of a causal process.

Taken together, the findings indicate two statistically coherent patterns rather than a confirmed dual-path process. One exploratory indirect association ran from perceived sequential organization through perceived route clarity to visit intention, indirect effect = 0.086. A second exploratory indirect association ran from perceived cinematic spatial cues through narrative transportation and emotional engagement, with the cinematic-cue → transportation → emotional-engagement indirect association = 0.109 and the transportation → emotional-engagement → visit-intention indirect association = 0.073. These patterns provide an integrated interpretation of the results while remaining bounded by the image-based, cross-sectional design.

### 5.3 Design implications

The findings point to several candidate designable spatial features as perceived through exhibition imagery. Sequential organization corresponds to perceivable starting points, transitions, focal nodes, and closure. Route clarity relates to entrance visibility, sightline continuity, directional cues, and spatial hierarchy. Cinematic spatial cues concern framing, visual focus, concealment–revelation, and spatial rhythm. These correspondences define possible links between designable spatial features and viewers’ perceptual responses, but they should be interpreted as exploratory perceptual associations rather than evidence that manipulating any single feature would change actual visitor movement, dwell time, or on-site behavior. The present study did not independently manipulate these features.

The findings suggest several provisional design implications. First, in perception-oriented exhibition planning and image-based evaluation, designers could attend to the structural organization of entrance, transition, focus, and closure so that visitors have clearer route judgments. This implication concerns perceived route clarity and should not be read as evidence that these features determine actual gallery movement.

Second, perceived cinematic spatial cues—such as concealment–revelation, framing, visual focus, and spatial rhythm—may offer a vocabulary for discussing how exhibition imagery organizes attention and emotional involvement. Because these cues were derived from a combined material-coding procedure and measured as perceptions, this implication is conceptual and design-oriented rather than evidence that any single cue independently produces a given response.

Third, the joint visit-intention model suggests that movement-related design may be considered not only in terms of traffic organization but also in relation to visitors’ perceived route clarity and emotional involvement. This implication concerns intended responses in image-based evaluation and should be tested with on-site behavioral measures before being generalized to actual visitation.

### 5.4 Limitations and future research

Several limitations warrant caution in interpreting the results.

First, and most importantly, the study measures perceived route clarity, not actual movement behavior. Image materials can reflect visitors’ subjective judgments of spatial guidance, but they are not equivalent to movement paths in a real gallery. Future work could combine on-site observation, indoor positioning, eye-tracking, or dwell-time data to test the correspondence between perceived route clarity and actual movement (Wang et al., 2025; Peponis et al., 2004). The viewing instruction asked respondents to imagine moving through the space. This may have strengthened route-clarity and narrative-transportation judgments, so future studies should compare alternative instructions and test whether the observed associations persist without an imagined-movement prompt.

Second, image stimuli cannot fully substitute for an on-site visit. The sense of scale, the multisensory atmosphere, and bodily presence in a real gallery are all difficult for two-dimensional images to reproduce. Future work could run A/B tests in real galleries or temporary exhibitions, or use immersive means such as VR for comparison, to test the ecological validity of these conclusions (Chang and Suh, 2025; Ariya et al., 2025; Bujić et al., 2023).

Third, the stimulus materials came from different museums and differed in theme, visual style, cultural background, and exhibit appeal, which may confound the results. Although topic interest, visual attractiveness, and familiarity were controlled, material heterogeneity cannot be fully ruled out. Consistent with this concern, the stimulus-variance-component sensitivity models showed that stimulus-level differences accounted for a substantial portion of outcome variance, with conditional *R*^2^ values near 0.72 (see Supplementary Table S3). The observed associations may therefore partly reflect material-specific features, such as museum type, image quality, theme familiarity, or visual style, rather than only the intended sequential or cinematic spatial cues. This concern is reinforced by the combined coding procedure and the limited pool of seven retained stimulus sets, which do not fully separate cue-level perceptions from stimulus-specific heterogeneity. The findings should therefore be interpreted as evidence from an image-based perceived-evaluation design rather than as general evidence about all museum exhibition spaces. Future studies should use a larger and more systematically balanced stimulus pool to distinguish cue-related associations from stimulus-specific heterogeneity. Moreover, copyright constraints prevent some stimuli from being fully released, which may affect reproducibility; future work could prioritize open-licensed images or secure authorization through museum collaboration.

Fourth, the measurement framework is preliminary: several focal constructs were adapted or newly worded for image-based evaluation, and the three covariates were single items. Although construct–item mapping was used, confirmatory validation was beyond this study. Future work should test the measurement structure with larger and more balanced stimulus pools.

Finally, although cluster-robust standard errors, linear mixed models, and bootstrap indirect-effect estimates were used as robustness checks, the dataset remains cross-sectional and same-source. These procedures strengthen the stability of the reported estimates but do not establish temporal order or causal effects; the mixed-model, cluster-robust, and bootstrap results should therefore be interpreted as convergent statistical evidence rather than confirmation of a causal mechanism. The respondent sample was also relatively young and highly educated, which limits generalization to broader museum visitor populations. Future studies should include more age-diverse and education-diverse samples, especially among on-site visitors.

## 6 Conclusion

This exploratory study examined how perceived sequential organization and cinematic spatial cues in museum-exhibition images relate to visitor experience and behavioral intention using a repeated participant-material analysis pipeline. Using participant-by-material online image-evaluation data, the analysis drew on 314 valid respondents, 918 valid material-level observations, and 830–852 complete-case primary mixed-model observations, yielding mixed-model coefficients from 0.234 to 0.374 and participant-clustered bootstrap indirect associations from 0.073 to 0.109.

The contribution lies chiefly in two respects. First, the study provides a preliminary operationalization of cinematic spatial cues as a questionnaire construct in museum exhibition-space psychology. Second, it shows how repeated participant-material evaluations can be analyzed with mixed models, clustered sensitivity checks, and bootstrap indirect-association estimates while keeping causal claims appropriately limited.

In practical terms, the study suggests that perceived sequential structure and cinematic spatial cues may be relevant to visitors’ subjective route clarity, transportation, emotional engagement, and visit intention in image-based evaluations. These implications should be tested in future studies using authorized stimuli, on-site behavioral measures, or experimental manipulations.

## Data Availability

The raw data supporting the conclusions of this article will be made available by the authors, without undue reservation.

## Supplementary material

SUPPLEMENTARY TABLE S1 Source and license metadata for the illustrative Figure 2 panels.

SUPPLEMENTARY TABLES S2-S11 Control-variable coefficients from the control-adjusted models, sensitivity-analysis outputs for the focal paths, sample-flow and exclusion summaries, discriminant-validity diagnostics, primary-model diagnostics, stimulus-coding scores, construct–item source mapping and questionnaire wording, material assignment and presentation rules, and participant-level manipulation-check results.
