# Supplementary material for: Museum exhibition-space psychology of sequential organization and cinematic spatial cues: an exploratory mixed-model association analysis
Source: Front Psychol. 2026 Oct 23;17:1939380. doi: 10.3389/fpsyg.2026.1939380 (PMC13739485; doi:10.3389/fpsyg.2026.1939380)
Supplement: Supplementary file 1 [file Table_1.DOCX]

# Supplementary Material

This file contains the supplementary information directly cited in the manuscript. It reports source/license metadata for the illustrative figure panels, theory-driven stimulus-coding records, construct–item source mapping, material assignment and presentation rules, participant-level manipulation checks, and compact computed summaries of the main sensitivity and diagnostic analyses. Raw response records, working spreadsheets, and analysis scripts are not reproduced here.

**Supplementary Table S1. Source and license metadata for the illustrative Figure 2 panels.**

| **Panel** | **Use** | **Illustrative category** | **File title** | **License** | **Credit** | **Source URL** |
| --- | --- | --- | --- | --- | --- | --- |
| A | Figure 2 | Sequential gallery axis | File:Victorian museum interior (Unsplash).jpg | CC0 | Kaiyu Wang marktwinkle | https://commons.wikimedia.org/wiki/File:Victorian_museum_interior_(Unsplash).jpg |
| B | Figure 2 | Open hall / low sequence | File:Museum of Science and Industry interior.jpg | CC0 | Reinhard Dietrich | https://commons.wikimedia.org/wiki/File:Museum_of_Science_and_Industry_interior.jpg |
| C | Figure 2 | Spatial rhythm | File:Statens Museum for Kunst, interior - Copenhagen - DSC08291.JPG | CC0 | Daderot | https://commons.wikimedia.org/wiki/File:Statens_Museum_for_Kunst,_interior_-_Copenhagen_-_DSC08291.JPG |
| D | Figure 2 | Framing / threshold | File:Interior - Princeton University Art Museum - DSC06695.jpg | Public domain | Daderot | https://commons.wikimedia.org/wiki/File:Interior_-_Princeton_University_Art_Museum_-_DSC06695.jpg |
| E | Figure 2 | Entrance sequence | File:Simose Art Museum entrance building interior.jpg | CC0 | Licjar Xeymelloz | https://commons.wikimedia.org/wiki/File:Simose_Art_Museum_entrance_building_interior.jpg |
| F | Figure 2 | Interior hall continuity | File:Interior of hall at CInema Museum, Kennington Lambeth.jpg | CC0 | Anonymous Unknown author | https://commons.wikimedia.org/wiki/File:Interior_of_hall_at_CInema_Museum,_Kennington_Lambeth.jpg |

*Note. These records document the copyright status of the illustrative panels used in Figure 2.*

**Supplementary Table S2. Control-variable coefficients from the control-adjusted sensitivity models.**

| **Model** | **Outcome** | **Control** | **beta** | **SE** | **z** | **p** | **CI low** | **CI high** | **N rows** |
| --- | --- | --- | --- | --- | --- | --- | --- | --- | --- |
| H1 | perceived_route_clarity | topic_interest | 0.056 | 0.0323 | 1.742 | 0.082 | -0.007 | 0.119 | 832 |
| H1 | perceived_route_clarity | visual_attractiveness | 0.060 | 0.0325 | 1.841 | 0.066 | -0.004 | 0.123 | 832 |
| H1 | perceived_route_clarity | familiarity | -0.020 | 0.0323 | -0.619 | 0.536 | -0.083 | 0.043 | 832 |
| H2 | narrative_transportation | topic_interest | 0.113 | 0.0328 | 3.454 | <0.001 | 0.049 | 0.178 | 823 |
| H2 | narrative_transportation | visual_attractiveness | 0.039 | 0.0329 | 1.172 | 0.241 | -0.026 | 0.103 | 823 |
| H2 | narrative_transportation | familiarity | 0.002 | 0.0328 | 0.055 | 0.956 | -0.062 | 0.066 | 823 |
| H3 | emotional_engagement | topic_interest | 0.076 | 0.0325 | 2.337 | 0.019 | 0.012 | 0.140 | 819 |
| H3 | emotional_engagement | visual_attractiveness | 0.015 | 0.0327 | 0.458 | 0.647 | -0.049 | 0.079 | 819 |
| H3 | emotional_engagement | familiarity | -0.018 | 0.0324 | -0.547 | 0.584 | -0.081 | 0.046 | 819 |
| H5/H6 | visit_intention | topic_interest | 0.034 | 0.0330 | 1.037 | 0.300 | -0.030 | 0.099 | 812 |
| H5/H6 | visit_intention | visual_attractiveness | 0.006 | 0.0329 | 0.189 | 0.850 | -0.058 | 0.071 | 812 |
| H5/H6 | visit_intention | familiarity | 0.070 | 0.0327 | 2.154 | 0.031 | 0.006 | 0.134 | 812 |

*Note. Coefficients are standardized estimates from participant-random-intercept LMMs that include the focal predictor(s) and the three control variables. These models are reported as sensitivity analyses because the control variables may be influenced by stimulus viewing.*

**Supplementary Table S3. Compact sensitivity summary for focal paths.**

| **Hyp.** | **Predictor** | **Adjusted beta** | **Adj. SE** | **Adj. 95% CI** | **N rows** | **VC beta** | **Stimulus var.** | **VC cond. R2** | **Cluster beta** | **Cluster SE** | **Cluster 95% CI** |
| --- | --- | --- | --- | --- | --- | --- | --- | --- | --- | --- | --- |
| H1 | perceived_sequential_organization | 0.348 | 0.032 | [0.285, 0.412] | 832 | 0.348 | 0.561 | 0.726 | 0.358 | 0.031 | [0.297, 0.419] |
| H2 | perceived_cinematic_spatial_cues | 0.324 | 0.033 | [0.26, 0.389] | 823 | 0.324 | 0.574 | 0.721 | 0.32 | 0.034 | [0.254, 0.387] |
| H3 | narrative_transportation | 0.361 | 0.033 | [0.297, 0.425] | 819 | 0.361 | 0.565 | 0.725 | 0.374 | 0.032 | [0.312, 0.435] |
| H5/H6 | emotional_engagement | 0.237 | 0.033 | [0.172, 0.301] | 812 | 0.237 | 0.565 | 0.725 | 0.234 | 0.035 | [0.167, 0.302] |

*Note. Adjusted models include the post-viewing control variables. VC models add stimulus variance components as a sensitivity diagnostic. Cluster models use participant-clustered robust standard errors. The stimulus variance-component sensitivity models changed the variance partitioning (see the elevated VC conditional R2 values) far more than the standardized fixed slopes; consequently, the adjusted and VC standardized focal coefficients are numerically very close and coincide at the reported three-decimal precision, even though they are estimated from two distinct models.*

**Supplementary Table S4. Sample-flow summary.**

| **Stage** | **N** | **Unit** | **Note** |
| --- | --- | --- | --- |
| Returned questionnaire records | 397 | participants | Initial records |
| Excluded at participant level | 83 | participants | See Supplementary Table S5 |
| Valid respondents | 314 | participants | 79.1% of returned records |
| Expected participant-material observations | 942 | rows | Three material evaluations per valid respondent |
| Removed at material level | 24 | rows | Material-level attention/completeness screening |
| Valid participant-material observations | 918 | rows | Rows entering scale-score analyses |
| Main LMM complete-case range | 830-852 | rows | Varies by outcome/predictor |
| All-item complete rows | 743 | rows | Rows for Harman/common-method diagnostic |

**Supplementary Table S5. Exclusion summary.**

| **Exclusion reason** | **n** | **% of returned** |
| --- | --- | --- |
| included | 314 | 79.093 |
| speeding | 21 | 5.29 |
| random_low_quality | 19 | 4.786 |
| attention_failed | 18 | 4.534 |
| straightlining | 14 | 3.526 |
| incomplete_viewing | 7 | 1.763 |
| excessive_missing | 4 | 1.008 |

*Note. Percentages are calculated against returned questionnaire records.*

**Supplementary Table S6. HTMT discrimination diagnostic summary.**

| **Diagnostic** | **Computed value** |
| --- | --- |
| Number of construct pairs | 15 |
| Item-complete N range | 743-743 |
| HTMT range | 0.155-0.649 |
| Highest HTMT pair | perceived_sequential_organization / perceived_cinematic_spatial_cues = 0.649 |
| Lowest HTMT pair | perceived_route_clarity / emotional_engagement = 0.155 |

*Note. The full pairwise matrix is summarized here because all values were below conventional conservative thresholds.*

**Supplementary Table S7. Primary participant-random-intercept diagnostics.**

| **Hyp.** | **Outcome** | **N rows** | **N participants** | **Participant var.** | **Residual var.** | **ICC** | **Marginal R2** | **Conditional R2** |
| --- | --- | --- | --- | --- | --- | --- | --- | --- |
| H1 | perceived_route_clarity | 852 | 313 | 0.027 | 0.845 | 0.031 | 0.128 | 0.155 |
| H2 | narrative_transportation | 843 | 314 | 0.015 | 0.883 | 0.016 | 0.103 | 0.118 |
| H3 | emotional_engagement | 840 | 314 | 0.023 | 0.837 | 0.027 | 0.14 | 0.163 |
| H5/H6 | visit_intention | 830 | 313 | 0.022 | 0.852 | 0.025 | 0.127 | 0.148 |

*Note. This table reports the compact computed diagnostics for the primary mixed models; raw model output is not reproduced.*

**Supplementary Table S8. Theory-driven coding scores for candidate stimulus sets.**

The six indicators were clarity of starting point, sequential continuity, concealment–revelation, visual focus, spatial rhythm, and sense of closure. Each indicator was scored from 0 to 2; preliminary grouping used totals of 0–4 (low), 5–8 (medium), and 9–12 (high).

| **Set ID** | **Museum/site label** | **Start** | **Continuity** | **Concealment–revelation** | **Visual focus** | **Spatial rhythm** | **Closure** | **Total** | **Preliminary level** | **Decision** |
| --- | --- | --- | --- | --- | --- | --- | --- | --- | --- | --- |
| H01 | Louvre | 2 | 2 | 2 | 2 | 2 | 1 | 11 | High | Retained |
| H02 | Vatican Museums | 2 | 2 | 2 | 2 | 1 | 1 | 10 | High | Retained |
| M03 | Rijksmuseum | 1 | 2 | 1 | 2 | 1 | 1 | 8 | Medium | Retained |
| M01 | Scottish National Gallery | 1 | 1 | 1 | 1 | 1 | 2 | 7 | Medium | Retained |
| M02 | National Museum of China | 1 | 1 | 1 | 1 | 1 | 1 | 6 | Medium | Retained |
| L02 | National Gallery of Ireland | 1 | 1 | 0 | 1 | 0 | 1 | 4 | Low | Retained |
| L01 | Metropolitan Museum of Art (Temple of Dendur) | 1 | 0 | 0 | 1 | 0 | 1 | 3 | Low | Retained |
| B01 | American Museum of Natural History | — | — | — | — | — | — | — | Backup | Excluded: object-heavy and less compatible with the required spatial sequence |

*Note. Ziqin Yuan and Chao Zhou independently coded all eight candidate sets without viewing one another’s scores. Discrepancies were reviewed item by item against the coding definitions and image sequences, and a final consensus coding result was established. No formal inter-rater reliability coefficient was calculated. B01 was excluded as a backup stimulus because its emphasis on object display was less compatible with the required exhibition-space sequence, route continuity, and narrative progression.*

**Supplementary Table S9. Questionnaire constructs, item IDs, source basis, study use.**

The questionnaire was administered in Chinese. The original Chinese wording is reproduced below. All items used a 7-point agreement scale (1 = strongly disagree, 7 = strongly agree) and were scored in the positive direction.

| Construct | Item ID | English wording | Original Chinese wording | Source basis | Use in the present study |
| --- | --- | --- | --- | --- | --- |
| Perceived sequential organization | psn_1 | This exhibition has a clear visiting sequence. | 这个展览具有清晰的参观顺序。 | Museum spatial/exhibition narrative research [1, 2] | Operationalizes perceived beginning-to-closure organization, continuity, focal development, and closure across an ordered exhibition-image sequence. |
| Perceived sequential organization | psn_2 | I can feel the exhibition content unfolding gradually like a story. | 我能感受到展览内容像故事一样逐步展开。 | Museum spatial/exhibition narrative research [1, 2] | Operationalizes perceived beginning-to-closure organization, continuity, focal development, and closure across an ordered exhibition-image sequence. |
| Perceived sequential organization | psn_3 | There is a coherent narrative relationship between the different exhibition areas or images. | 不同展区或画面之间存在连贯的叙事关系。 | Museum spatial/exhibition narrative research [1, 2] | Operationalizes perceived beginning-to-closure organization, continuity, focal development, and closure across an ordered exhibition-image sequence. |
| Perceived sequential organization | psn_4 | I can identify the beginning, development, focal point, and closure of this exhibition. | 我能判断这个展览中的开端、发展、重点和收束部分。 | Museum spatial/exhibition narrative research [1, 2] | Operationalizes perceived beginning-to-closure organization, continuity, focal development, and closure across an ordered exhibition-image sequence. |
| Perceived cinematic spatial cues | csc_1 | This exhibition directs my gaze toward important exhibits or core areas. | 这个展览通过视线引导让我注意到重点展项或核心区域。 | Architecture–film spatial theory and museum environmental-psychology research [1, 3] | Operationalizes perceived framing, visual focus, concealment–revelation, spatial rhythm, and scene progression in exhibition imagery. |
| Perceived cinematic spatial cues | csc_2 | This space has clear concealment-and-revelation effects. | 这个空间具有明显的遮挡与揭示效果。 | Architecture–film spatial theory and museum environmental-psychology research [1, 3] | Operationalizes perceived framing, visual focus, concealment–revelation, spatial rhythm, and scene progression in exhibition imagery. |
| Perceived cinematic spatial cues | csc_3 | The spatial rhythm of this exhibition gives me a feeling similar to cinematic camera movement. | 这个展览的空间节奏让我产生类似镜头推进的感觉。 | Architecture–film spatial theory and museum environmental-psychology research [1, 3] | Operationalizes perceived framing, visual focus, concealment–revelation, spatial rhythm, and scene progression in exhibition imagery. |
| Perceived cinematic spatial cues | csc_4 | This exhibition has a strong sense of scene and visual imagery. | 这个展览具有较强的场景感和画面感。 | Architecture–film spatial theory and museum environmental-psychology research [1, 3] | Operationalizes perceived framing, visual focus, concealment–revelation, spatial rhythm, and scene progression in exhibition imagery. |
| Perceived route clarity (PMG item block) | pmg_1 | I can clearly determine where I should begin visiting this exhibition. | 我能清楚判断应该从哪里开始参观这个展览。 | Museum layout, path organization, and accessibility research [4] | Operationalizes inferred starting point, route clarity, spatial focus, and willingness to follow the represented direction. |
| Perceived route clarity (PMG item block) | pmg_2 | This exhibition makes me feel that the visiting route is clear. | 这个展览让我感到参观路线是清晰的。 | Museum layout, path organization, and accessibility research [4] | Operationalizes inferred starting point, route clarity, spatial focus, and willingness to follow the represented direction. |
| Perceived route clarity (PMG item block) | pmg_3 | I can identify which areas or exhibits are the main points of the visit. | 我能判断哪些区域或展项是参观重点。 | Museum layout, path organization, and accessibility research [4] | Operationalizes inferred starting point, route clarity, spatial focus, and willingness to follow the represented direction. |
| Perceived route clarity (PMG item block) | pmg_4 | I am willing to continue visiting in the direction presented by this exhibition. | 我愿意沿着这个展览呈现的方向继续参观。 | Museum layout, path organization, and accessibility research [4] | Operationalizes inferred starting point, route clarity, spatial focus, and willingness to follow the represented direction. |
| Narrative transportation | nt_1 | I am attracted by the content presented in this exhibition. | 我被这个展览呈现的内容吸引。 | Narrative transportation theory and the Transportation Scale–Short Form [5, 6] | Adapts attention, imagery, and narrative involvement to ordered museum-exhibition images. |
| Narrative transportation | nt_2 | While viewing these materials, I can imagine myself entering the situation presented by this exhibition. | 观看这些材料时，我能想象自己进入了这个展览所呈现的情境。 | Narrative transportation theory and the Transportation Scale–Short Form [5, 6] | Adapts attention, imagery, and narrative involvement to ordered museum-exhibition images. |
| Narrative transportation | nt_3 | While viewing these materials, I feel that I am following a narrative thread. | 观看这些材料时，我感到自己跟随了一条叙事线索。 | Narrative transportation theory and the Transportation Scale–Short Form [5, 6] | Adapts attention, imagery, and narrative involvement to ordered museum-exhibition images. |
| Narrative transportation | nt_4 | This exhibition makes me interested in learning more about how the story develops. | 这个展览让我产生继续了解故事发展的兴趣。 | Narrative transportation theory and the Transportation Scale–Short Form [5, 6] | Adapts attention, imagery, and narrative involvement to ordered museum-exhibition images. |
| Emotional engagement | ee_1 | This exhibition creates an emotional connection for me. | 这个展览让我产生情感连接。 | Museum emotional-engagement and EMOTIVE research [2] | Operationalizes emotional connection, empathy, personal relevance, interest, and emotional resonance. |
| Emotional engagement | ee_2 | I can empathize with the people, history, or cultural themes presented in the exhibition. | 我能对展览中的人物、历史或文化主题产生共情。 | Museum emotional-engagement and EMOTIVE research [2] | Operationalizes emotional connection, empathy, personal relevance, interest, and emotional resonance. |
| Emotional engagement | ee_3 | The content of this exhibition is related to my personal experiences, values, or interests. | 这个展览内容与我的个人经验、价值或兴趣产生关联。 | Museum emotional-engagement and EMOTIVE research [2] | Operationalizes emotional connection, empathy, personal relevance, interest, and emotional resonance. |
| Emotional engagement | ee_4 | This exhibition makes me feel interested, moved, or emotionally resonant. | 这个展览让我感到兴趣、感动或情绪共鸣。 | Museum emotional-engagement and EMOTIVE research [2] | Operationalizes emotional connection, empathy, personal relevance, interest, and emotional resonance. |
| Visit intention | vi_1 | If I have the opportunity, I would like to visit this exhibition or the related museum in person. | 如果有机会，我愿意实地参观这个展览或相关博物馆。 | Digital-museum experience and visit-intention research [7, 8] | Operationalizes stated intention to visit, recommend, or learn more about the represented exhibition or museum. |
| Visit intention | vi_2 | I would recommend this exhibition or the related museum to others. | 我愿意向他人推荐这个展览或相关博物馆。 | Digital-museum experience and visit-intention research [7, 8] | Operationalizes stated intention to visit, recommend, or learn more about the represented exhibition or museum. |
| Visit intention | vi_3 | I would like to learn more about this exhibition or related topic. | 我愿意进一步了解这个展览或相关主题。 | Digital-museum experience and visit-intention research [7, 8] | Operationalizes stated intention to visit, recommend, or learn more about the represented exhibition or museum. |
| Topic interest | topic_interest | I am interested in the topic of this exhibition itself. | 我对这个展览的主题本身感兴趣。 | Museum visitor-experience and digital-museum research [7, 8] | Sensitivity covariate measuring interest in the exhibition topic. |
| Visual attractiveness | visual_attractiveness | This set of materials is visually attractive. | 这组材料在视觉上具有吸引力。 | Museum visitor-experience and digital-museum research [7, 8] | Sensitivity covariate measuring perceived visual attractiveness of the image set. |
| Familiarity | familiarity | Before viewing the questionnaire materials, I was already familiar with this museum, exhibition, or related topic. | 在观看问卷前，我已经熟悉这个博物馆、展览或相关主题。 | Museum visitor-experience and digital-museum research [7, 8] | Sensitivity covariate measuring prior familiarity with the museum, exhibition, or topic. |

*Note. Source basis and development status are reported at the item-set level because items within each construct were developed or adapted from the same conceptual source base. Content validity was assessed through construct–dimension–item mapping and item-by-item checks of content coverage, clarity for general respondents, construct relevance, and redundancy. No independent external expert-panel review, cognitive interview, or participant pretest was conducted. Ziqin Yuan prepared the initial Chinese translation; Chao Zhou independently reviewed and back-translated the items. The English source, Chinese version, and back-translation were compared item by item, and discrepancies were resolved through discussion. Construct scores were calculated as the arithmetic mean of the relevant items.*

**Supplementary Table S10. Material assignment, presentation, and randomization.**

| **Procedure component** | **Implementation in the formal questionnaire study** |
| --- | --- |
| Material levels per respondent | Each respondent evaluated one high-level, one medium-level, and one low-level material set. |
| Within-level assignment | The available sets were H01/H02 for high, M01/M02/M03 for medium, and L01/L02 for low. These options generated 12 possible high-medium-low combinations, all of which were observed among the 314 respondents retained at the questionnaire level. Their participant-level frequencies are reported below. |
| Order of the three blocks | The high-, medium-, and low-level blocks were presented in randomized order. |
| Order within a selected set | Each selected set contained six images presented in the fixed sequence predefined for that set. |
| Order of evaluation and attention items | Evaluation items and attention-check items were presented in fixed order; item randomization was not used. |

**Realized high-medium-low material combinations among the 314 questionnaire-level valid respondents.**

| **Combination** | **High set** | **Medium set** | **Low set** | **n** |
| --- | --- | --- | --- | --- |
| 1 | H01 | M01 | L01 | 24 |
| 2 | H01 | M01 | L02 | 22 |
| 3 | H01 | M02 | L01 | 21 |
| 4 | H01 | M02 | L02 | 31 |
| 5 | H01 | M03 | L01 | 27 |
| 6 | H01 | M03 | L02 | 21 |
| 7 | H02 | M01 | L01 | 34 |
| 8 | H02 | M01 | L02 | 37 |
| 9 | H02 | M02 | L01 | 29 |
| 10 | H02 | M02 | L02 | 27 |
| 11 | H02 | M03 | L01 | 22 |
| 12 | H02 | M03 | L02 | 19 |

*Note. The assignment and presentation rules describe the participant–material structure used in the dataset. They do not imply that the high, medium, and low groups independently manipulated narrative and cinematic cues; the levels were formed through the combined theory-driven material-coding procedure described in Supplementary Table S8.*

**Supplementary Table S11. Participant-level manipulation-check results across material levels.**

| **Construct** | **Low n** | **Low M (SD)** | **Medium n** | **Medium M (SD)** | **High n** | **High M (SD)** | **Wald χ² (df = 2)** | **p** | **Participant ICC** |
| --- | --- | --- | --- | --- | --- | --- | --- | --- | --- |
| Perceived sequential organization | 297 | 3.87 (0.82) | 291 | 4.82 (0.82) | 299 | 5.62 (0.77) | 833.23 | <0.001 | 0.163 |
| Perceived cinematic spatial cues | 299 | 4.01 (0.81) | 296 | 4.98 (0.83) | 290 | 5.75 (0.77) | 801.07 | <0.001 | 0.132 |

*Note. The participant-random-intercept mixed-model checks showed the expected high > medium > low pattern for both perceived sequential organization and perceived cinematic spatial cues. These results support perceived differentiation across the material levels, but because the levels were formed through a combined coding procedure, they do not establish fully independent manipulation of the two cue dimensions.*

**Note.** The supplementary measurement references use the independent numbering within the supplementary material; the bibliographic format follows the manuscript reference list. Newly worded indicates that item wording was formulated for the present image-based context from the cited conceptual literature; it does not denote a literature-free or independently validated new scale. The route-clarity block retains the dataset code PMG for traceability.

**References for questionnaire measures (independent supplementary numbering)**

1. Zhang, Z., Lu, J., and Zhang, X. (2024). Emotionally-oriented design in museums: a case study of the Jewish Museum Berlin. Front. Psychol. 15:1423466. doi: 10.3389/fpsyg.2024.1423466

2. Economou, M., Young, H., and Sosnowska, E. (2025). Digital storytelling for emotional engagement in museums: design and evaluation of the Hunterian Antonine Wall EMOTIVE experiences. Int. J. Herit. Stud. 32, 324–355. doi: 10.1080/13527258.2025.2591613

3. Kafili, D., and Brennan, A. (2025). Enactive sight: the intersection of architectural space and cinematic camerawork in videogame visualisations. Vis. Stud. 1–24. doi: 10.1080/1472586X.2025.2560643

4. Wang, X., et al. (2025). Natural movement: measuring optimal pathway configuration of museum layout and pedestrian accessibility through visibility graph analysis and intelligent agent simulation. Front. Archit. Res. 14, 1228–1248. doi: 10.1016/j.foar.2025.03.001

5. Green, M. C., and Brock, T. C. (2000). The role of transportation in the persuasiveness of public narratives. J. Pers. Soc. Psychol. 79, 701–721. doi: 10.1037/0022-3514.79.5.701

6. Appel, M., Gnambs, T., Richter, T., and Green, M. C. (2015). The Transportation Scale–Short Form (TS-SF). Media Psychol. 18, 243–266. doi: 10.1080/15213269.2014.987400

7. Meng, W., and Dolah, J. (2025). From virtual museum experience quality to offline visit intention: a cultural identity mediation model for sustainable heritage engagement. Sustainability 17:10664. doi: 10.3390/su172310664

8. Deng, Y., et al. (2023). From digital museuming to on-site visiting: the mediation of cultural identity and perceived value. Front. Psychol. 14:1111917. doi: 10.3389/fpsyg.2023.1111917

Note on image-guided imagined progression: Before each image set, respondents were instructed to view the six images in order and to imagine moving through the space. This instruction concerns image-guided imagined progression and does not measure physical movement, observed routes, or dwell time in an actual exhibition space.
